# Novel Biphasic Role of LipoxinA_**4**_ on Expression of Cyclooxygenase-2 in Lipopolysaccharide-Stimulated Lung Fibroblasts

**DOI:** 10.1155/2011/745340

**Published:** 2011-07-02

**Authors:** Shengxing Zheng, Qian Wang, Qian He, Xiaorong Song, Duyun Ye, Fang Gao, Shengwei Jin, QingQuan Lian

**Affiliations:** ^1^Department of Anaesthesia, Second Affiliated Hospital of WenZhou Medical College, Zhejiang 325003, China; ^2^Department of Pathophysiology, Tongji Medical College, Huazhong University of Science and Technology, 13 Hangkong Road, Wuhan, Hubei Province 430030, China; ^3^Warwick Medical School, University of Warwick, Coventry CV4 7AL, UK; ^4^Academic Department of Critical Care, Resuscitation, Anaesthesia, and Pain, Heart of England NHS Foundation Trust, Birmingham B9 5SS, UK

## Abstract

Fibroblasts are important to host defence and immunity, can also as initiators of inflammation as well. As the endogenous “braking signal”, Lipoxins can regulate anti-inflammation and the resolution of inflammation. We investigated the effect of lipoxinA_4_ on the expression of cyclooxygenase-2 in lipopolysaccharide-stimulated lung fibroblasts. We demonstrated that the expression of cyclooxygenase-2 protein was significantly increased and peaked initially at 6 hours, with a second increase, with maximal levels occurring 24 hours after lipopolysaccharide challenge. ProstaglandinE_2_ levels also peaked at 6 hours, and prostaglandinD_2_ levels were increased at both 6 and 24 hours. Exogenous lipoxinA_4_ inhibited the first peak of cyclooxygenase-2 expression as well as the production of prostaglandinE_2_ induced by lipopolysaccharide in a dose-dependent manner. In contrast, exogenous lipoxinA_4_ increased the second peak of cyclooxygenase-2 expression as well as the production of prostaglandinD_2_ induced by lipopolysaccharide in a dose-dependent manner. LipoxinA_4_ receptor mRNA expression was markedly stimulated by lipopolysaccharide but inhibited by lipoxinA_4_. We present evidence for a novel biphasic role of lipoxinA_4_ on the expression of cyclooxygenase-2 in lipopolysaccharide-stimulated lung fibroblasts, whereby LXA_4_ has an anti-inflammatory and proresolving activity in lung fibroblasts following LPS stimulation.

## 1. Introduction

The acute inflammatory reaction in the lung is a complex response but is usually self-limiting and resolves. Traditionally, immune effector cells such as lymphocytes and macrophages have been considered to have a fundamental role in the development of inflammation. Traditionally, fibroblasts have only been considered as a structural element. Recent studies, however, demonstrate that pulmonary fibroblasts, far from being merely bystander cells, are important to host defence but may also promote lung injury. Recent evidence has shown that fibroblasts can produce proinflammatory cytokines and prostaglandins (PGs) and can act as initiators of inflammation as well as regulators of immunity [1–3]. When activated, fibroblasts are capable of producing inflammatory mediators, including interleukin-8 (IL-8), monocyte chemoattractant protein-1, express cyclooxygenase-2 (COX-2), with the resultant release of proinflammatory PGs such as protaglandinE_2_ (PGE_2_) [3]. Moreover, fibroblasts can be directly activated by exposure to lipopolysaccharide (LPS) [4]. Gram-negative bacteria can be responsible for the failure of early treatment and significantly increased morbidity and mortality in patients with pneumonia [5, 6]. LPS, as a major constituent of Gram-negative bacterial cell walls, is recognized by the innate immune system of cells, such as resident tissue fibroblasts [7]. Specifically, Toll receptor expression on these fibroblasts may be able to direct the tissues' response to injury to promote inflammation resolution.

 Cyclooxygenase is a key enzyme that catalyzes the conversion of arachidonicacid to prostaglandin [8]. There are two cyclooxygenase isoforms, COX-1 and COX-2. COX-1 is produced constitutively in most cell types, whereas COX-2 is inducible [8]. Prostaglandins are lipid mediators synthesized from arachidonic acid by the actions of COX enzymes [9]. They can be secreted by resident fibroblasts, as well as inflammatory cells, in response to TNF-*α*, IL-1*β*, or LPS [10, 11]. Prostaglandins also contribute to the signs and symptoms of inflammation [12]. ProstaglandinE_2_, the main PG produced during inflammatory response, is a proinflammatory lipid mediator of inflammation and participates in initiation of inflammation [13]. Previous studies suggest PGD_2_, as a proresolution mediator, also actively contributes to the resolution of tissue injury and inflammation [14]. 

 Lipoxins (LXs) are trihydroxytetraene-containing eicosanoids mainly formed through transcellular biosynthesis involving either 5- and 15-lipoxygenases (LOXs) or 5- and 12-lipoxygenases (LOXs) as well as COX-2 in respiratory tissues [15, 16]. Lipoxins were the first proresolving mediators to be recognized; they not only have anti-inflammatory properties, but also promote the resolution of inflammation [17]. Lipoxins have been described as the endogenous “braking signal” for inflammation [18–20]. Lipoxins have been extensively studied in asthma [21, 22], cystic fibrosis [23–25], and in various infections. These studies have highlighted LXs as potential novel therapeutic agent for the treatment of inflammatory disease. 

 A recent study reported that injured bronchial epithelial cells upregulated lipoxinA_4_ (LXA_4_) receptor (LXA_4_R) in a COX-2-dependent manner to promote LXA_4_-mediated resolution of airway inflammation [26]. In response to acid injury, epithelial cells rapidly increased COX-2 and PGE_2_ expression [26]. The COX-2 enzyme has also been implicated as an important mediator of pulmonary fibrosis, with COX-2^−/−^ mice having increased fibrotic lung responses [27]. More recently, Medeiros et al. reported that LXA_4_ also repressed the expression and activity of COX-2 on endotoxin-induced uveitis (EIU) in rats [28]. Moreover, LXA_4_ has also been shown to inhibit connective tissue growth factor- (CTGF) stimulated proliferation of human lung fibroblasts [25], and fibroblasts directly simulated by LPS are capable of producing COX-2 and PGE_2 _[3, 4]. However, the time course of COX-2 expression in lung fibroblasts stimulated by LPS and the effect of LXA_4_ on expression of COX-2 PGE_2_ and PGD_2_ remain unclear.

 In this study, we examined the expression of COX-2 and the production of PGE_2_ and PGD_2_ in lung fibroblasts after LPS challenge. Additionally, we also investigated the effect of LXA_4_ on the expression of COX-2 and the production of PGE_2_ and PGD_2_. Finally, we investigated the effect of LXA_4_ or LPS on LXA_4_R mRNA expression in lung fibroblasts; we present evidence for a novel biphasic role of LXA_4_ on expression of COX-2 in LPS-stimulated lung fibroblasts.

## 2. Materials and Methods

### 2.1. Materials

LipoxinA_4_, from Cayman Chemical Company, was stored at −80°C until being diluted in serum-free culture medium immediately before use. Lipopolysaccharide (LPS; E. coil serotype 055 : B5) was purchased from Sigma. DMEM, FCS, Trypsin EDTA, and enzyme-free cell dissociation buffer were purchased from Gibco. Penicillin and streptomycin in saline citrate buffer were from Invitrogen. Hoechst 33258 was obtained from Novus. Anti-CD31, anti-Vimentin and anti-COX-2 were purchased from Abcam. Anti-Cytokeratin-8 and anti-F4/80 were purchased from Santa Cruz.

### 2.2. Cell Culture

Rat pulmonary fibroblasts were isolated from Sprague-Dawley rats as previously described [29]. Lung tissue was cut into <1-mm^3^ pieces and dissociated in Hanks buffered saline solution (HBSS) containing 0.25% trypsin at 37°C for 1.5 min. Trypsin was inhibited by DMEM with 15% fetal calf serum (FCS) and dissociated tissue centrifuged at 1000 g for 5 minutes at 4°C. The dissociated tissue pieces were placed into a culture plate with DMEM containing 15% FCS and left to allow fibroblast outgrowth. After fibroblasts had grown out from the tissues, usually 2–3 days, the remaining tissue was removed by aspiration, and the cells were allowed to reach confluence. Confluent fibroblasts were then passaged with a split ratio of 1 : 2 by trypsin treatment and used for the experiments at passages 3–5. The purity of fibroblast cultures were consistently over 99% as established morphologically by their typical spindle shape and characteristics and by expression of the fibroblast marker vimentin and negative expression of endothelial (CD31), macrophage (F4/80), and epithelial (cytokeratin-8) cell markers. 

 For all experiments, cells were subcultured into six-well plates and maintained until subconfluence (80%), confluent cells (100%) were serum deprived for 24 hours with low-serum medium (DMEM supplemented with 0.1% FCS) prior to the addition of LPS and/or LXA_4_. The cells were then incubated with LPS (1 *μ*g/mL) for 6, 12, 24, 48, and 72 hours. For LXA_4_ experiments, the cells were incubated in the low-serum medium containing 1 *μ*g/ml LPS in the presence or absence of 100, 200, or 400 nmol/mL of LXA_4_ for 6 or 24 hours.

### 2.3. Haematoxylin and Eosin (H&E) Staining

Fibroblasts grow in culture on coverslips were fixed with 4% paraformaldehyde in PBS for 10 min, stained with Haematoxylin (BDH, Lutterworth, UK) for 10 minutes, incubated in Scott's tap water (tap water with a few drops of 1 M sodium hydroxide) for 5 min and stained with alcoholic Eosin solution for 5 min. They were washed by immersion in tap water for 2 minutes after every step. Cells were then differentiated by immersion in 0.1% hydrochloric acid-ethanol each for 30 s and mounted by inversion onto glass slides dotted with Gel/Mount. Images were taken by an inverted microscope (IX70, Olympus America, Inc., Melville, NY, USA) with a 1.40NA 60 × objective sets. Image size was 2560 × 1920.

### 2.4. Indirect-Immunofluorescence

Fibroblasts were grown to approximately 70% confluence on poly-D-lysine-coated glass coverslips in 24-well plates, fixed with 4% paraformaldehyde in PBS for 10 minutes, rinsed three times with PBS, and permeabilized by 0.2% Triton X-100/PBS for 2 minutes followed by 0.5% Triton X-100 (Pierce) in PBS for 10 minutes. Nonspecific binding of antibodies was prevented by the addition of 5% bovine serum albumin in PBS for 30 minutes at 37°C. The samples were then incubated overnight at 4°C with antivimentin (1 : 100), anti-CD31 (1 : 100), anti-F4/80 (1 : 100) or anti-Cytokeratin-8 (1 : 200) in 2% BSA/PBS. Following three PBS washes, cells were incubated for 2 hours with fluorescein-conjugated immunopure goat anti-mouse IgG (H+L) or goat antirabbit IgG (H+L) (1 : 200) respectively, in 5% BSA/PBS at room temperature. After washing three times with PBS, cell nuclei were counter stained with Hoechst (1 : 1000) for 15 minutes, followed by three PBS washes. Cells were then mounted by inversion onto glass slides dotted with Gel/Mount. Images were taken by an inverted microscope (IX70, Olympus America, Inc., Melville, NY) with a 1.40NA 60 × objective and FITC, rhodamine and Cy5 filter sets. Image size was  2560 × 1920.

### 2.5. PGE_2_ and PGD_2_ Protein Expression

Fibroblast supernatants were collected following treatments, centrifuged (1500 g, 5 minutes), aliquoted, and stored at −80°C. PGE_2_ and PGD_2_ protein expressionwas measured by ELISA according to the manufacturer's instructions (R&D systems). Assays were run in triplicate and repeated twice.

### 2.6. COX-2 Protein Expression

Fibroblasts were lysed and homogenized in 200 *μ*L of cold lysis buffer (150 mM NaCl, 1% Triton X-100, 1% sodium deoxycholate, 0.1% SDS, 50 mmol/L Tris-HCl (pH 7.2), 0.2 mM sodium vanadate, 1% phenylmethylsulfonyl fluoride, and 0.2% aprotinin). Samples were incubated on ice for 20 minutes and then centrifuged at 12,000 rpm for 10 minutes. Protein concentrations of the supernatants were determined by using a BCA protein assay (Pierce). Proteins were separated in 10% SDS polyacrylamide gels and transferred onto nitrocellulose membranes. Expression of COX-2 was determined using primary rabbit anti-COX-2 antibody (1 : 750) and secondary horseradish peroxidase-conjugated goat antirabbit IgG. Protein expression of *β*-actin served as a loading control. The bound antibody was detected by enhanced chemiluminescence on an X-ray film.

### 2.7. RNA Isolation, Reverse Transcription and PCR

Total RNA was extracted using TriZol reagent (Life Technologies) followed by phenol-chloroform extraction and ethanol precipitation (Fisher Scientific). RNA purity was checked by spectrophotometry, and RNA integrity was confirmed by visualization of 28 S and 18 S bands on an agarose gel. 1 *μ*g of RNA was reverse transcribed using avian myeloblastosis virus reverse transcriptase (Promega). PCR analysis was performed with the following sets of primers: for rat LXA_4_ receptor 5′-TGTTGGGCCCTGGATTTTAGC-3′ (sense) and 5′-TGTTACCCCAGGATGCGAAGTT-3′ (antisense), amplifying a 116-bp fragment [30] and for *β*-actin, used as an internal control 5′-AACAGTCCGCCTAGAAGCAC-3′ (sense) and 5′-CGTTGACATCCGTAAAGACC-3′ (antisense), generating a 281-bp fragment. PCR for the rat LXA_4_ receptor consisted of 35 repetitive cycles of predenaturing at 95°C for 4 minutes, denaturing at 94°C for 30 seconds, annealing at 59°C for 40 seconds, extension at 72°C for 40 seconds, and a final extension at 72°C for 5 minutes. For r-actin an annealing temperature of 56°C was used. Amplified cDNA was separated on a 1.6% agarose gel and visualized using ethidium bromide. Semiquantitative analysis was performed using UVP-gel densitometry (SanGabriel, Calif. USA). 

## 3. Results

### 3.1. Purification and Identification of Primary Lung Fibroblasts

**Figure 1:**
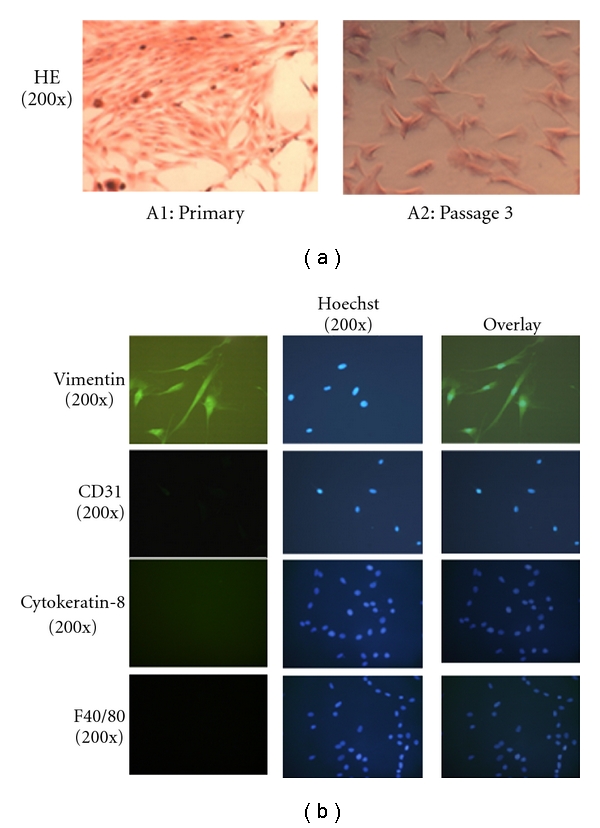
Purification and identification of primary lung fibroblasts. (a) Untreated fibroblasts isolated from rat lungs were stained with hematoxylin and eosin for conventional morphological evaluation under light microscope (Nikon eclipse 90i, Tokyo, Japan). Primary cultures (A1), still contained some non-fibroblasts cells. At passage 3 (A2), fibroblast purity was consistently over 99% as established morphologically by their typical spindle shape and characteristics. (b) Cultures were stained by indirect-immunofluorescence assay for Vimentin, CD31, cytokeratin-8, or F4/80. Cultures contained only Vimentin positive cells indicative of fibroblasts.

Untreated fibroblast were stained with hematoxylin and eosin for conventional morphological evaluation under light microscope (Nikon eclipse 90i, Tokyo, Japan) (Figure 1(a)) or stained by indirectimmunofluorescence for Vimentin, CD31, Cytokeratin-8 and F4/80 expressions (Figure 1(b)). Vimentin was used as marker of fibroblast cells, CD31 as marker of endothelial cells [31], F4/80 as a surface marker of macrophages [32, 33], and Cytokeratin-8, as a marker of epithelial cells [34]. We observed only cells with fibroblast morphology which stained only for Vimentin, therefore, only purified fibroblasts were cultured.

### 3.2. The Effect of LPS on COX-2, PGE_2_ and PGD_2_ Expression in Lung Fibroblasts

**Figure 2:**
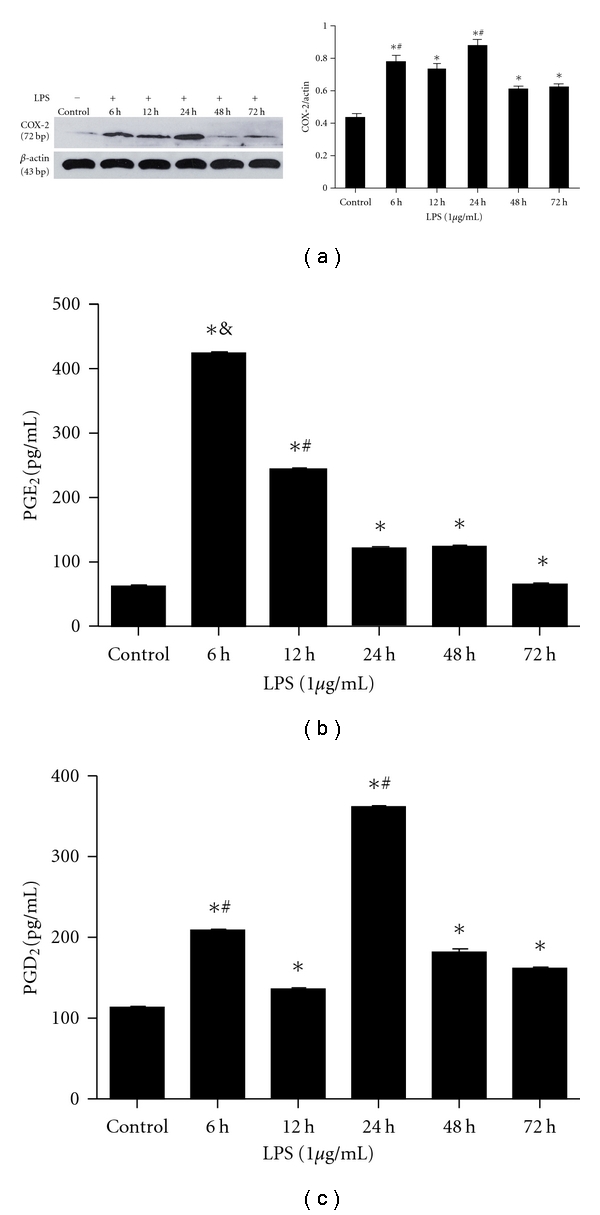
The effect of LPS on COX-2, PGE_2_, and PGD_2_ expression in lung fibroblasts. (a) Rat lung fibroblasts were incubated with LPS (1 *μ*g/mL) for 6, 12, 24, 48, and 72 hours. The expression of COX-2 protein was assessed by western blot and analysed by densitometry compared to *β*-actin expression. COX-2 expression peaked initially at 6 hours, and then with maximal levels at 24 hours after-LPS treatment (*P < 0.05 versus non-LPS group ^#^ *P* < 0.05 versus 12, 48, 72 hours groups). (b) Supernatants were collected after LPS (1 *μ*g/mL) treatment for 6, 12, 24, 48 and 72 hours. PGE_2_ protein was measured by ELISA. Data are expressed as mean ± SE for each group. (*P < 0.05 versus non-LPS group; & *P* < 0.01 versus 12, 24, 48, and 72 hours groups; ^#^ *P* < 0.05 versus 6, 24, 48, and 72 hours groups). (c): Supernatants were collected after LPS (1 *μ*g/mL) treatment for 6, 12, 24, 48 and 72 hours. PGD_2_ protein was measured by ELISA. Data are expressed as mean ± SE for each group. (*P < 0.05 versus non-LPS group; ^#^ *P* < 0.05 versus 12, 48, 72 hours groups).

To determine the dynamic expression of COX-2 in rat lung fibroblasts, our isolated fibroblasts were incubated with LPS (1 *μ*g/mL) for 6, 12, 24, 48, and 72 hours. The expression of COX-2 protein was significantly increased and peaked initially 6 hours after LPS stimulation, with maximal levels occurring at 24 hours (Figure 2(a)). In contrast, PGE_2_ levels were increased only at 6 hours (Figure 2(b)), with the precursor of prostaglandin J series, PGD_2_ [35], levels increased at both 6 and 24 hours. (Figure 2(c)).

### 3.3. The Effect of LXA_4_ on LPS-Induced Expression of COX-2 Protein Expression and PGE_2_, and PGD_2_ Production at 6 Hours in Primary Lung Fibroblasts

**Figure 3:**
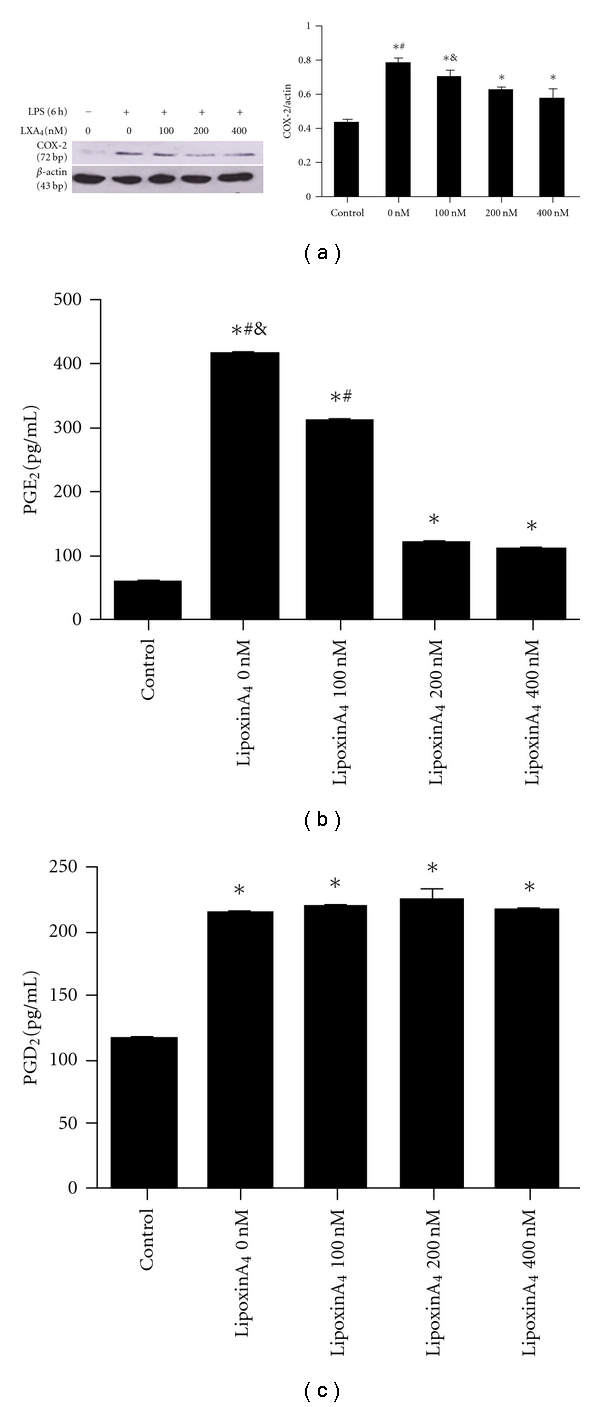
The effect of LXA_4_ on LPS-induced expression of COX-2 protein expression and PGE_2_ and PGD_2_ production at 6 hours in primary lung fibroblasts. (a) Rat lung fibroblast cells were treated with LXA_4_ at 0, 100, 200 or 400 nM in the presence of LPS (1 *μ*g/mL) for 6 hours. Cells were then harvested, sonicated and COX-2 protein detected by western blot. *P < 0.05 versus control group, ^#^ *P* < 0.05  versus (100, 200 or 400 nM) LXA_4_ groups; & *P* < 0  .05 versus (200 or 400 nM) LXA_4_ groups. (b) Supernatants from rat lung fibroblast cells treated with LXA_4_ at 0, 100, 200, or 400 nM in the presence of LPS (1 *μ*g/mL) for 6 hours were collected and PGE_2_ protein measured by ELISA. Data are expressed as mean ± SE for each group. *P < 0.05 versus control group, ^#^ *P* < 0.05  versus (100, 200, or 400 nM) LXA_4_ groups; & *P* < 0.05 versus (200 or 400 nM) LXA_4_ groups. (c) Supernatants from rat lung fibroblast cells treated with LXA_4_ at 0, 100, 200 or 400 nM in the presence of LPS (1 *μ*g/mL) for 6 hours were collected and PGD_2_ protein measured by ELISA. Data are expressed as mean ± SE for each group. *P < 0.05 versus control group.

To determine whether exogenous LXA_4_ modulates COX-2 expression after LPS stimulation, we reassessed COX-2 protein at 6 hours with various concentrations of LXA_4_ treatment in our isolated lung fibroblasts. Using LXA_4_ at 100, 200, or 400 nmol/ml we observed inhibition of COX-2 protein expression in a dose-dependent manner (Figure 3(a)). Moreover, after cells were incubated with LXA_4_ for 6 hours, PGE_2_ and PGD_2_ protein levels in the supernatant were measured by ELISA (Figure 3(b) and 3(c), resp.). PGE_2_ secretion was inhibited by LXA_4_ in a dose-dependent manner, decreasing from 411.734 ± 1.364 pg/mL in 0 nM LXA_4_ treated cells to 307.075 ± 2.151 pg/mL in 100 nM LXA_4_-treated fibroblasts and then further still to 108.089 ± 4.851 pg/mL in 400 nM LXA_4_-treated fibroblasts (*P* < 0.05). In contrast although PGD_2_ levels, increased with LXA_4_ treatement, this was not observed to be dose dependent.

### 3.4. The Effect of LXA_4_ on LPS-Induced Expression of COX-2 Protein Expression and PGE_2_ and PGD_2_ Production at 24 Hours in Primary Lung Fibroblasts

**Figure 4:**
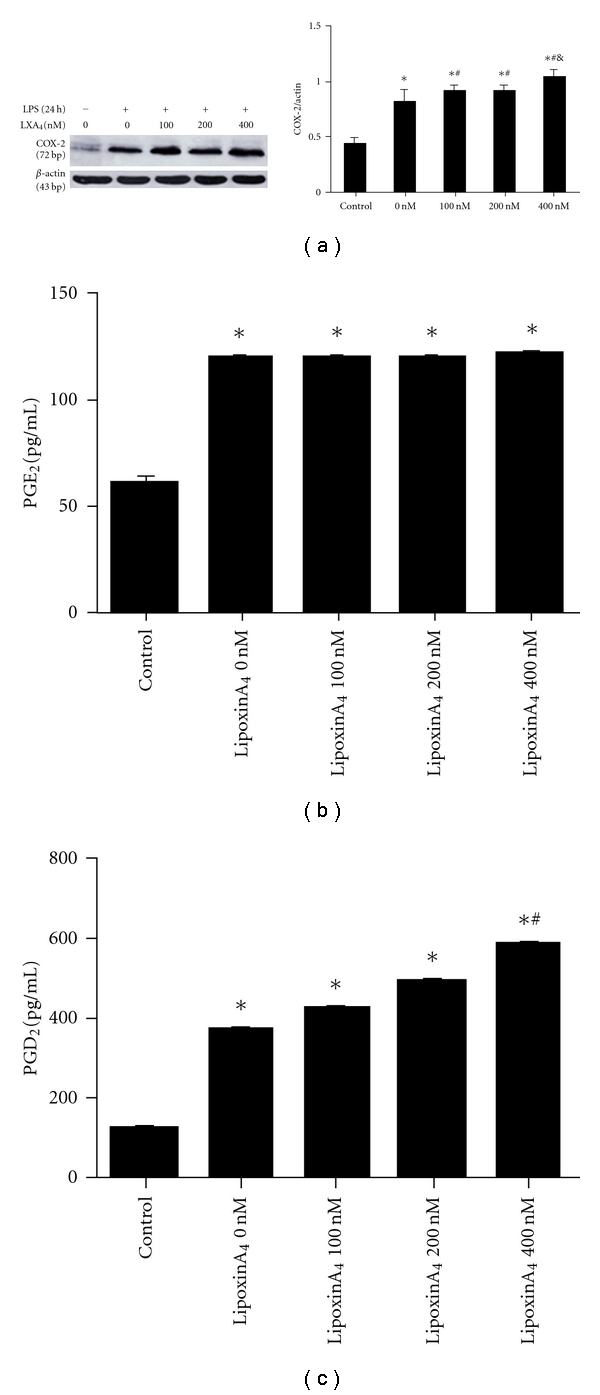
The effect of LXA_4_ on LPS-induced expression of COX-2 protein expression and PGE_2_ and PGD_2_ production at 24 hours in primary lung fibroblasts. (a) Rat lung fibroblast cells were treated with LXA_4 _ at 0, 100, 200, or 400 nM in the presence of LPS (1 *μ*g/mL) for 24 hours. Cells were then harvested, sonicated, and COX-2 protein detected by Western blot. *P < 0.05 versus control group, ^#^ *P* < 0.05 versus non-LXA_4_ groups; & *P* < 0.05 versus (100 or 200 nM) LXA_4_ groups. (b) Supernatants from rat lung fibroblast cells treated with LXA_4_ at 0, 100, 200, or 400 nM in the presence of LPS (1 *μ*g/mL) for 24 hours were collected and PGE_2_ protein measured by ELISA. Data are expressed as mean ±  SE for each group. *P < 0.05 versus control group. (c) Supernatants from rat lung fibroblast cells treated with LXA_4_ at 0, 100, 200 or 400 nM in the presence of LPS (1 *μ*g/mL) for 24 hours were collected and PGD_2_ protein measured by ELISA. Data are expressed as mean ± SE for each group.*P < 0.05 versus control group, ^#^ *P* < 0.05  versus (0, 100 or 200 nM) LXA_4_ groups.

To determine whether treatment with exogenous LXA_4_ affected the secondary increase of COX-2 expression after LPS stimulation, we also reassessed COX-2 protein at 24 hours after various concentrations of lipoxinA4 treatment. Using LXA_4_ at 100, 200, or 400 nmol/mL, we observed an increase in COX-2 protein expression in a dose-dependent manner (Figure 4(a)). We also measures secretion of PGE_2_ and PGD_2_ following LPS and LXA_4_ treatments (Figures 4(b) and 4(c), resp.). Interestingly, in contrast to our result at 6 hr LPS treatment although levels of PGE_2_ increased with LXA_4_, it was not dose-dependent. Furthermore, PGD_2_ secretion following this treatment regime was enhanced by LXA_4_ in a dose-dependent manner, increasing from 367.170 ± 4.773 pg/mL in 0 nM LXA_4_-treated cells to 417.916 ± 3.251 pg/mL following 100 nM LXA_4_-treated fibroblasts and 584.307 ± 15.478 pg/mL in 400 nM LXA_4_-treated fibroblasts (*P* < 0.05).

### 3.5. LipoxinA_4_ Receptor is Expressed in Rat Lung Fibroblasts and Upregulated by LPS

**Figure 5:**
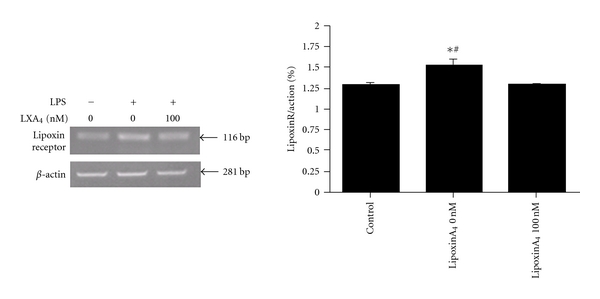
LipoxinA_4_ receptor is expressed in rat lung fibroblasts and upregulated by LPS. Rat lung fibroblast cells were treated with LPS (1 *μ*g/mL) in the presence or absence of LXA_4 _(100 nM) of for 24 hours. mRNA was isolated and semiquantiative RT-PCR for LXA_4_R and *β*-actin were performed. Amplified cDNA was separated by 1.6% agarose gel electrophoresis, visualized with ethidium bromidem and analyzed by gel densitometry relative to *β*-actin expression. Lane 1: untreated control. Lane 2: LPS only (1 *μ*g/mL). Lane 3: LPS (1 *μ*g/mL) and LXA_4_ (100 nM). *P < 0.05 versus control group, ^#^ *P* < 0.05  versus (100 nM) LXA_4_ group. These data represent 6 individual experimental repeats.

LipoxinA4 interactions with its receptor, LXA_4_R, play a significant role in regulating leukocyte functions [36]. Therefore, we tested whether LXA_4_R mRNA expression altered following LPS and LXA_4_ treatment in rat lung fibroblasts by using semiquantitative RT-PCR. A single band corresponding to LXA_4_R mRNA expression was amplified (Figure 5, lane 1), which when analysed by densitometry was markedly stimulated by LPS treatment (Figure 5, lane 2). Interestingly, we observed that cotreatment with LPS and LXA_4_ reduced expression of LXA_4_R back to that seen in untreated controls (Figure 5, lane 3).

## 4. Discussion

Acute lung injury (ALI)/ARDS is an inflammatory lung disease with high mortality [36–39]. Treatment of inflammatory diseases today is largely based on interrupting the synthesis or action of mediators that also decrease the host's ability to successfully deal with infection, given that the innate inflammatory response is a beneficial defensive event [40]. Recently, resolution of acute inflammation was shown to be an active, rather than a passive process, and endogenous chemical mediators play key roles in its programmed resolution and returning to homeostasis [41]. Among them, lipoxins and aspirin-triggered lipoxins evoke bioactions in a range of physiologic and pathophysiologic processes and serve as endogenous lipid/chemical mediators that stop neutrophilic infiltration and initiate resolution [17]. Development of strategies that promote the resolution of inflammation is a novel therapeutic measure to attenuate inflammatory lung injury. 

 In a previous study, we clearly demonstrated that post-treatment with lipoxinA_4_ (LXA_4_) significantly reduces LPS- induced ALI in mice [42]. Lipoxin also promotes gradual resolution of fibrosis in lung [43]. In addition, LXA_4_ repressed the expression and the activity of COX-2 on endotoxin-induced uveitis in rats [28]. In carrageenin-induced pleurisy in rats, COX-2 protein expression peaked initially at 2 hours, and at 48 hours, there was a second increase in COX-2 expression in inflammatory cells separated from the inflammatory exudates [44]. Taking these data together, our purpose was to find out whether COX-2 expression in lung fibroblasts stimulated by LPS also has two peaks and, if so, how LXA_4_ affect the expression of COX-2 and the production of prostaglandins, specifically PGE_2_ and PGD_2_.

 Our data clearly demonstrated that the expression of COX-2 protein was significantly increased and peaked initially 6 hours after LPS stimulation in lung fibroblasts. This was also associated with maximal PGE_2_ synthesis, However, following 24 hours of LPS stimulation, there was a second increase in COX-2 expression, this time associated with maximal PGD_2_ synthesis. Thus, as inflammation progresses into resolution, PGE_2_ synthesis declines, giving way to a prominence of COX-2-derived PGD_2_, both of which play important roles in mediating resolution. This data indicates that COX-2 may be proinflammatory (via PGE_2 _ expression) during the development of inflammation, but anti-inflammatory (via PGD_2_ expression) during resolution in lung fibroblasts. Recent studies have also highlighted a role for COX-2-derived PGs serving anti-inflammatory and anti-fibrotic roles in the resolution of inflammation [13, 45, 46]. In a model of spontaneously resolving ALI, selective COX-2 inhibition results in prolonged inflammation, in part, by decreasing production of pro-resolving mediators, including LXA_4_ and 15-epimer-LXA_4 _[44, 45]. So, a late, anti-inflammatory effect of COX-2, instead of the more widely appreciated early, proinflammatory action, was crucial to the timely recovery from ALI [45]. 

 Our results also demonstrated that the expression of COX-2 as well as PGE_2_ production by fibroblast cells was significantly inhibited by LXA_4_ in a dose-dependent manner 6-hour LPS treatment, suggesting that LXA_4 _has a potential anti-inflammatory role in lung fibroblasts during the onset of inflammation. Consistent with our findings, similar results have shown that LXA_4 _also repressed the expression and the activity of COX-2 on endotoxin-induced uveitis in rats [28]. Interestingly, the expression of COX-2 by fibroblast cells of its second increase (24 hours) was significantly promoted by LXA_4_ in a dose-dependent manner. In addition, and consistent with the results above, LXA_4_ inhibited the production of PGE_2_ while promoted the production of PGD_2_ in the supernatants. Therefore, our study demonstrates a novel biphasic role of LXA_4 _ on the expression of COX-2 and the production of PGE_2_ and PGD_2_, suggesting that LXA_4_ has a potential anti-inflammatory and proresolving roles in LPS-stimulated lung fibroblasts.

 As the endogenous “braking signals” in inflammation [18–20], lipoxins are produced locally in the lung to regulate inflammatory cells. Furthermore, the specific receptor with high affinity for LXA_4_ (LXA_4_R) has been cloned from myeloid lineages [15, 16]. Expression of LXA_4_R is required to evoke actions of lipoxins in each tissue therefore, the receptor expression can also control biological function of lipoxins *in vivo*. LXA_4_R belongs to the G-protein coupled receptor superfamily of proteins and is widely distributed in cells and tissues [15]. Our results indicate clearly for the first time that LXA_4_R mRNA was expressed in rat lung fibroblasts and is upregulated by LPS stimulation. Moreover, possibly due to a negative feedback mechanism, LXA_4_R mRNA was inhibited by cotreatment of LPS and LXA_4_.

 In summary, this study demonstrated that COX-2 protein expression peaks initially at 6 hours but then also at 24 hours after LPS stimulation in isolated lung fibroblasts. Moreover, LXA_4_ has a novel biphasic role on expression of COX-2 and production of PGE_2_ and PGD_2_, whereby LXA_4_ has an anti-inflammatory and proresolving activity in lung fibroblasts following LPS stimulation. Therefore, our study may provide a novel target for future therapies for controlling LPS-induced ALI.

## Abbreviations

LPS: Lipopolysaccharide
COX-2: Cyclooxygenase-2
PGE_2_: ProstaglandinE_2_
PGD_2_: ProstaglandinD_2_
PGs: Prostaglandins
LXA_4_: LipoxinA_4_
LXA_4_R: LipoxinA_4_ receptor.
